# An Elevated Body Mass Index Increases Lung Volume but Reduces Airflow in Italian Schoolchildren

**DOI:** 10.1371/journal.pone.0127154

**Published:** 2015-05-13

**Authors:** Fabio Cibella, Andreina Bruno, Giuseppina Cuttitta, Salvatore Bucchieri, Mario Raphael Melis, Stefano De Cantis, Stefania La Grutta, Giovanni Viegi

**Affiliations:** 1 National Research Council of Italy, Institute of Biomedicine and Molecular Immunology, Palermo, Italy; 2 Department of Economics, Statistics, and Business Sciences—University of Palermo, Palermo, Italy; University of Tennessee Health Science Center, UNITED STATES

## Abstract

**Background:**

Asthma and obesity are important and growing health issues worldwide. Obesity is considered a risk factor for asthma, due to the induction of changes in airway mechanics and altered airway inflammation.

**Methods:**

We cross-sectionally investigated the effect of increased weight on pulmonary function in a large population sample of healthy children, aged 10–17 yrs living in Palermo, Italy. Explanatory effect of weight on lung function variables were evaluated by multiple linear regression models, taking into account height, gender, and age-class.

**Results:**

Among the 2,393 subjects, FVC and FEV_1_ were positively correlated to weight. Multiple regression models showed that the weight beta coefficient for FEV_1_ was significantly lower with respect to that for FVC (0.005 and 0.009 l/kg, respectively), indicating a different magnitude in explanatory effect of weight on FVC and FEV_1_. Both FEV_1_/FVC and FEF_25–75%_/FVC ratios were negatively correlated to weight, while FEF_25–75%_ was not significantly correlated. Similar results were obtained also when 807 symptomatic subjects were introduced in the model through a sensitivity analysis.

**Conclusion:**

In healthy children, the disproportionate increase of FEV_1_ and FVC with weight produces airflow decrease and consequently apparent poorer lung function independently from respiratory disease status.

## Introduction

Asthma and obesity are two important and growing health issues in industrialized countries. The prevalence of overweight children in the United States is continuing to increase, having risen from 6.5% to approximately 19% in schoolchildren between 1976–1980 and 2003–2004 [1], while the prevalence of adolescent obesity has nearly tripled in the last 2 decades [2]. This issue is not limited to the United States but involves many other Western and developing countries as well [3,4]. A recent report from the American Thoracic Society indicates that obesity is a risk factor for asthma in all evaluated demographic groups and that factors which could contribute to asthma pathogenesis in obese individuals include both airway mechanics and altered inflammation and immune responses related to being obese [5].

Numerous cross-sectional studies have reported that the prevalence of asthma is higher in obese versus lean individuals. Particularly, in a recent cross-sectional study conducted on 5,351 children aged 4–18 years, obesity was associated with a higher prevalence of asthma in children, with no evidence of significant modulation by either gender or age [6]. Furthermore, prospective studies, performed on both children and adults, have indicated that obesity antedates asthma and that weight-loss is associated with substantial improvements in the clinical status, lung function, symptoms, and asthma control in severe asthmatics with high body mass index (BMI) [7]. On the other hand, a recent prospective study investigating 6–15 year-old asthmatics concluded that the observed relationships between respiratory function with BMI are not specific of asthma, and being overweight is not associated with significant clinical impacts on asthma during childhood [8]. In addition, another prospective study performed in both children and adults found that there was no relationship in children between BMI and severity of asthma, spirometry findings, quality of life, or health care utilization [9]. Similarly, we have recently demonstrated that being overweight-obese does not have a significant effect on airway inflammation as measured by exhaled nitric oxide in adolescents [10].

Furthermore, when the relationship between body fat and lung function was evaluated in subjects of both genders without respiratory diseases, inconsistent results were obtained [11,12].

Therefore, the present study was aimed at investigating the effect produced by weight on pulmonary function in healthy children through the evaluation of a large sample of children previously enrolled in two cross-sectional surveys and all included in a narrow age range [13,14].

## Materials and Methods

Two cross sectional studies were performed on random samples of white Caucasian schoolchildren, aged 10–17 years, living in the city of Palermo, in the Mediterranean area of southern Italy: the first one in 2004, on 1,050 children from 8 schools [13], the second one in 2005–2006, on 2,150 children from 16 schools [14].

Self-administered questionnaires were completed by adolescents at school, regarding past and current respiratory allergic symptoms and personal information. A child's history of “wheeze ever” was defined as a positive answer to the question “*Have you ever had wheezing or whistling in your life*”. A history of nocturnal cough and post-exercise cough or wheeze was also investigated. Information on possible confounders or effect modifiers was also collected. Exposure to mould/dampness at home was evaluated using the question: “*Have you ever seen mould/dampness/fungi on the walls or on the ceiling of your bedroom*”. Current passive smoking exposure (ETS) was assessed through the question “*Are there smokers at home*”. Self-reported traffic exposure was recorded as the frequency of trucks passing on the street of residence on weekdays (*never/rare/frequent/constant*), and subjects were considered exposed if they answered ‘frequent’ or ‘constant’.

Height and weight were measured in all the children in a standing position without shoes, using a stadiometer and an electronic digital scale: BMI was computed as weight/height^2^ (kg/m^2^). Overweight (OW) and obese (O) children were defined following the gender- and age-specific cut-off points for overweight and obese by Cole et al [15]. Pulmonary function tests were performed through a portable spirometer (MicroLoop, Micro Medical, Chatham Maritime, Kent, UK). Forced expiratory volume in one second (FEV_1_), forced vital capacity (FVC) and maximum mid-expiratory flow (FEF_25–75%_) were measured according to ATS/ERS guidelines [16]: the best FVC and FEV_1_ were retained and FEF_25–75%_ was selected from the manoeuvre with the largest sum of FEV_1_ and FVC. Spirometric predicted values were those from Quanjer et al [17]. For each subject, FEV_1_/FVC and FEF_25–75%_/FVC ratios were also computed.

### Statistics

A total of 3,200 children were enrolled, of which 807 reporting wheeze ever, nocturnal cough, or exercise-induced cough were excluded from the initial analyses: thus, 2,393 healthy subjects were evaluated.

Absolute values of FVC, FEV_1_, and FEF_25–75%_, along with absolute FEV_1_/FVC and FEF_25–75%_/FVC ratios were introduced into multiple linear regression models (as dependent variables) and tested against sex, age (as age classes: ≤11, 12, 13, and ≥14 years), weight, height (i.e., the two components of BMI). One-way analysis of variance (ANOVA) was used for testing differences between means. The possible association between categorical variables was evaluated by χ^2^ test.

### Ethics Statement

Both the epidemiological studies were approved by the Ethics Committee of the University Hospital of Palermo. All parents of the invited schoolchildren signed a written informed consent. According to Italian law, respect of individual privacy concerning clinical data was guaranteed.

## Results

None of the investigated children declared him/herself to be an active smoker. The initial evaluation was performed on 2,393 healthy subjects: there were 654 (27.3%) OW and 279 (11.7%) O. General characteristics of the study sample are presented in Table 1. The prevalence of OW and O subjects was significantly higher among boys. The characteristics of the children reporting respiratory symptoms and thus excluded from the analysis are reported in Table 2: spirometric indices of airflow limitation showed that pulmonary function was significantly poorer in symptomatic rather than asymptomatic subjects, whereas no difference was found in BMI. Symptomatic subjects showed an increased frequency of domestic exposure to ETS, mould/dampness, and self-reported heavy traffic exposure. The prevalence of OW and O among symptomatic subjects was 28.0% and 13.0% respectively, not significantly different from that found in asymptomatic individuals (Table 2).

**Table 1:** General characteristics of the study sample composed by asymptomatic children, separately for males and females (No. = 2,393).

	Males	Females
No. (%)	1,171 (48.9)	1,222 (51.1)
Age (yrs, mean [range])	12.5 (10–17)	12.4 (10–16)
FVC % pred (mean [SD])	96.9 (±11.7)	96.6 (±11.6)
FEV_1_% pred (mean [SD])	100.6 (±11.9)	100.3 (±11.4)
FEF_25–75%_ % pred (mean [SD])	104.1 (±21.5)	103.0 (±21.2)
FEV_1_/FVC (%, mean [SD])	88.9 (±5.6)	92.0 (±5.4)
FEF_25–75%_/FVC (L/s/L, mean [SD])	1.06 (±0.23)	1.19 (±0.27)
BMI (kg/m^2^, mean [SD])	21.6 (±4.5)	21.0 (±4.2)
BMI class.: Non obese-non overweight children (No. [%])	662 (56.5)	798 (65.3)*
Overweight children (No. [%])	333 (28.5)	321 (26.3)*
Obese children (No. [%])	176 (15.0)	103 (8.4)*
Mould/dampness exposure (No. [%])	147(12.5)	171(13.9)
Current passive smoking exposure (No. [%])	637(54.4)	704(57.6)
Self-reported heavy traffic exposure (No. [%])	214(18.3)	258(21.1)

*p<0.0001, χ^2^.

In BMI classification, overweight and obese children were defined following the gender- and age-specific cut-off points by Cole et Al[15]

Otherwise indicated, differences were not significant.

**Table 2:** Characteristics of enrolled sample (No. = 3,200).

	Asymptomatic (No. = 2,393)	Symptomatic (No = 807)	p value
Male/Female (No.)	1,171/1,222	375/432	0.25*
BMI (mean [±SD])	21.3(±4.34)	21.5(±4.25)	0.37**
BMI class.: Non-overweight/non-obese (No, %)	1,460(61.0%)	476(59.0%)	0.52*
Overweight (No, %)	654(27.3%)	226(28.0%)	
Obese (No, %)	279(11.7%)	105(13.0%)	
FVC (% pred, mean [±SD])	96.8(±11.7)	97.4(±11.5)	0.17**
FEV_1_ (% pred, mean [±SD])	100.4(±11.7)	99.5(±11.3)	0.065**
FEF_25–75%_ (% pred, mean [±SD])	103.5(±21.4)	99.1(±21.2)	<0.0001**
FEV_1_/FVC (%, mean [±SD])	90.5(±5.68)	89.1(±6.09)	<0.0001**
FEF_25–75%_/FVC (L/s/L, mean [±SD])	1.12(±0.26)	1.07(±0.26)	<0.0001**
Mould/dampness exposure (No. [%])	318(13.3)	135(16.7)	0.025*
Current passive smoking exposure (No. [%])	1,341(56.0)	524(64.9)	<0.0001*
Self-reported heavy traffic exposure (No. [%])	472(19.7)	210(26.0)	0.0002*

*χ^2^ test

**one-way-ANOVA.

In BMI classification, overweight and obese children were defined following the gender- and age-specific cut-off points by Cole et Al[15]

Data are shown separately for asymptomatic subjects (No. = 2,393) for multiple regression analysis (Tables 3 and 4) and symptomatic individuals (No. = 807) included in the analysis presented in Tables 7 and 8.

**Fig 1:**
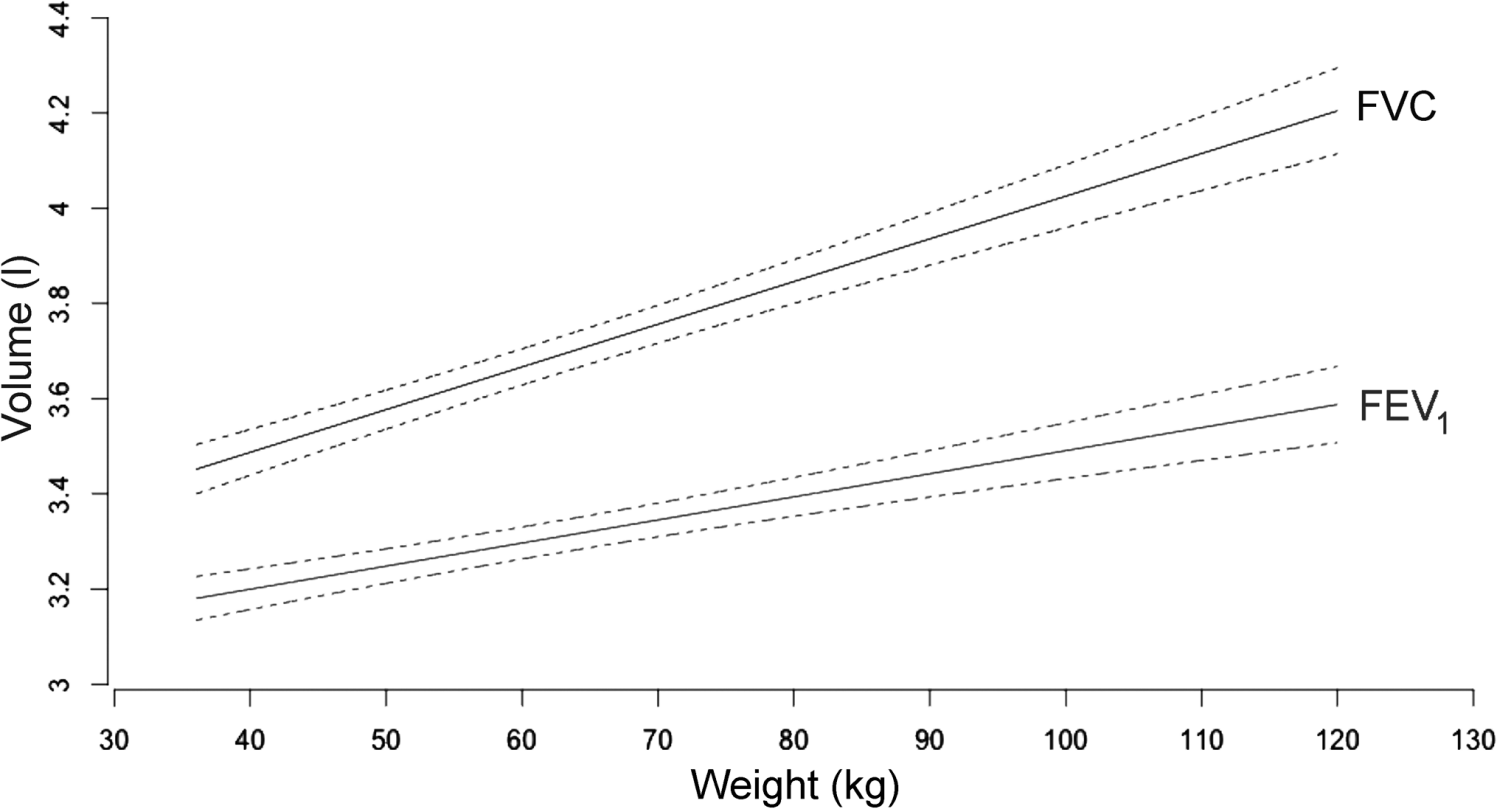
Slopes of FVC and FEV_1_ linear regression lines to weight. Linear regression lines (i.e., the fitted values followed by the lower and upper bounds of the 95% confidence interval for mean response) of the relationships between Forced Vital Capacity (FVC) and Forced Expiratory Volume in One second (FEV_**1**_), as response variables, and weight, as explanatory one, plotted according to parameters estimates in Table 3, for a male child belonging to the ≥14 yrs old age class, of 1.64m height (mean value for male 14 yrs old subjects in the sample).

In healthy children, the available variables potentially acting as confounding factors or effect modifiers were included in a multiple regression model. A backward stepwise selection process allowed us to extrapolate the only variables with a significant effect on respiratory indices, excluding domestic ETS, mould/dampness, and self-reported traffic exposures, thus identifying a “minimal” set including sex, age, height, and weight. Moreover, interactions between variables were tested, but none of them were determined to be significant. In Tables 3 and 4, R^2^ values and the β coefficients (with 95% confidence intervals) are provided for the multiple linear regression models with either FVC, FEV_1_, and FEF_25–75%_ (Table 3) or FEV_1_/FVC and FEF_25–75%_/FVC (Table 4) as dependent variables. When controlling for sex, age, and height, weight showed significant and positive β coefficients for both FVC and FEV_1_: it is noteworthy that the weight β coefficient for FVC was higher than the weight β coefficient for FEV_1_. While the weight β coefficient was not significant for FEF_25–75%_, those relevant to FEV_1_/FVC and FEF_25–75%_/FVC were negative and significant. To confirm these results and avoid any age effect, we performed the same analysis stratifying the regression model by age classes. The results confirmed the significant weight effect within each age class (Table 5): furthermore, the weight β coefficient for FVC was always higher than the weight β coefficient for FEV_1_ and those for FEV_1_/FVC and FEF_25–75%_/FVC were always negative (with the exception of the weight β coefficient for FEV_25–75%_/FVC, which was not significant in the ≤11 years age class). In Fig 1 a graphical representation of the FVC/weight and FEV_1_/weight relationships is presented: the regression lines (along with relevant 95% confidence intervals) were produced using coefficients from Table 3 for a male child belonging to the ≥14 years age class of 1.64m height (the mean height of males in that age class in the studied sample).

**Table 3:** Parameter estimated by multiple linear regression analysis models for FVC, FEV_1_, and FEF_25–75%_ as dependent variables and sex, age class, height, and weight as independent variables.

Dependent var.	FVC, R^2^ = .669			FEV_1_, R^2^ = .660			FEF_25–75%_, R^2^ = .341		
Parameter	β coefficient*	p value	95% confidence interval	β coefficient*	p value	95% confidence interval	β coefficient*	p value	95% confidence interval
Intercept	-4.230	.000	-4.576/-3.884	-3.886	.000	-4.193/-3.579	-4.167	.000	-4.837/-3.496
[Gender = F]	-.178	.000	-.207/-.149	-.071	.000	-.096/-.046	.166	.000	.110/.221
[Gender = M]	0^a^	.	.	0^a^	.	.	0^a^	.	.
[Age class ≤ 11]	-.137	.000	-.191/-.083	-.200	.000	-.248/-.152	-.438	.000	-.543/-.333
[Age class = 12]	-.108	.000	-.153/-.063	-.159	.000	-.199/-.119	-.330	.000	-.418/-.243
[Age class = 13]	-.033	.141	-.077/.011	-.076	.000	-.115/-.037	-.169	.000	-.255/-.084
[Age class ≥ 14]	0^a^	.	.	0^a^	.	.	0^a^	.	.
Height (m)	4.487	.000	4.248/4.726	4.202	.000	3.991/4.414	4.987	.000	4.525/5.450
Weight (kg)	.009	.000	.008/.010	.005	.000	.004/.006	-.001	.467	-.004/.002

^a^This parameter is set to zero because it is redundant

*β coefficients indicate how much a dependent variable changes per each unit variation of the independent variable, taking into account the effect of the other independent variables in the model. For categorical variables, β coefficients represent the effect of moving from the “reference” category (^a^) to another

Data are shown for 2,393 asymptomatic children.

**Table 4:** Parameter estimated by multiple linear regression analysis models for FEV_1_/FVC and FEF_25–75%_/FVC as dependent variables and sex, age class, height, and weight as independent variables.

Dependent var.	FEV_1_/FVC, R^2^ = .128			FEF_25–75%_/FVC, R^2^ = .101		
Parameter	β coefficient*	p value	95% confidence interval	β coefficient*	p value	95% confidence interval
Intercept	88.096	.000	82.913/93.279	1.295	.000	1.056/1.534
[Gender = F]	3.023	.000	2.594/3.452	.125	.000	.105/.144
[Gender = M]	0^a^	.	.	0^a^	.	.
[Age class ≤ 11]	-2.599	.000	-3.407/-1.791	-.104	.000	-.141/-.067
[Age class = 12]	-1.912	.000	-2.588/-1.236	-.071	.000	-.102/-.040
[Age class = 13]	-1.395	.000	-2.054/-.736	-.046	.003	-.076/-.015
[Age class ≥ 14]	0^a^	.	.	0^a^	.	.
Height (m)	4.993	.006	1.419/8.567	.006	.940	-.158/.171
Weight (kg)	-.104	.000	-.124/-.083	-.004	.000	-.005/-.003

^a^This parameter is set to zero because it is redundant

*β coefficients indicate how much a dependent variable changes per each unit variation of the independent variable, taking into account the effect of the other independent variables in the model. For categorical variables, β coefficients represent the effect of moving from the “reference” category (^a^) to another

Data are shown for 2,393 asymptomatic children.

**Table 5:** β coefficients* estimated by multiple linear regression analysis for FVC, FEV_1_, FEV_25–75%_, FEV_1_/FVC, and FEV_25–75%_/FVC as dependent variables and sex, height, and weight as independent variables, separately for each age class (≤11, 12, 13, and ≥14 years), among 2,393 asymptomatic children.

Age class	≤11 years		12 years		13 years		≥14 years	
Dependent var.	β coefficient	95%CI	β coefficient	95%CI	β coefficient	95%CI	β coefficient	95%CI
FVC	**0.007**	0.004/0.010	**0.011**	0.009/0.013	**0.008**	0.005/0.010	**0.009**	0.005/0.012
FEV_1_	**0.004**	0.001/0.007	**0.007**	0.005/0.009	**0.004**	0.002/0.006	**0.004**	0.001/0.007
FEV_25–75%_	0.001	-0.005/0.008	0.001	-0.003/0.006	-0.003	-0.008/0.002	-0.003	-0.010/0.003
FEV_1_/FVC	**-0.081**	-0.143/-0.019	**-0.109**	-0.147/-0.072	**-0.099**	-0.134/-0.064	**-0.115**	-0.160/-0.070
FEV_25–75%_/FVC	-0.002	-0.005/0.000	**-0.004**	-0.006/-0.002	**-0.004**	-0.005/-0.002	**-0.004**	-0.006/-0.002

*β coefficients indicate how much a dependent variable changes per each unit variation of the independent variable, taking into account the effect of the other independent variables in the model. For categorical variables, β coefficients represent the effect of moving from the “reference” category (^a^) to another

Only the β coefficients relevant to weight are shown, with 95% confidence interval (95%CI). Significant β coefficients are presented in bold.

When mean values of lung function variables were analyzed in OW and O categories, FEV_1_ and FVC (in percent of predicted) were significantly higher in both OW and O sub-samples (male and female, while no difference was found for FEF_25–75%_ (Table 6). Conversely, FEV_1_/FVC and FEF_25–75%_/FVC (absolute percent) were lower in both male and female OW and O subjects (Table 6). As a sensitivity analysis, we performed the same comparisons on the whole sample also including the 807 children reporting wheeze ever or nocturnal cough or exercise-induced cough, for a total of 3,200 subjects (Tables 7 and 8). In this model, the presence/absence of allergic respiratory diseases was introduced as an independent variable: intercepts and weight β coefficients for FVC, FEV_1_, FEV_1_/FVC, FEF_25–75%_, and FEF_25–75%_/FVC remained substantially unchanged. The β coefficients for presence of respiratory symptoms were significant for FEV_1_, FEV_1_/FVC, FEF_25–75%_, and FEF_25–75%_/FVC. Among symptomatic children, the differences in mean lung function values among non OW-O, OW and O subjects were quite similar to those found in asymptomatic individuals (Table 9).

**Table 6:** Analysis performed on 2,393 asymptomatic children.

	Males (No. = 1,171)				Females (No. = 1,222)			
	Non OW-O, (No. = 662)	OW, (No. = 333)	O, (No. = 176)	p value	Non OW-O, (No. = 798)	OW-O, (No. = 321)	O, (No. = 103)	p value
FVC (% of predicted, mean[SD])	95.5(11.5)	98.3(11.9)	99.5(11.6)	<0.0001	94.4(11.4)	100.2(10.5)	102.8(12.0)	<0.0001
FEV_1_ (% of predicted, mean[SD])	99.8(11.6)	101.6(12.5)	101.6(11.9)	0.04	98.6(11.5)	103.2(10.4)	104.1(11.4)	<0.0001
FEF_25–75%_ (% of predicted, mean[SD])	104.3(21.7)	104.7(21.6)	102.2(20.9)	0.42	102.7(21.5)	103.7(20.3)	103.2(21.8)	0.75
FEV_1_/FVC (%, mean[SD])	89.6(5.7)	88.3(5.0)	87.0(5.6)	<0.0001	92.7(5.3)	91.1(5.1)	89.7(5.6)	<0.0001
FEF25-75%/FVC(L/s/L, mean[SD])	1.08(0.24)	1.04(0.21)	1.00(0.22)	<0.0001	1.22(0.28)	1.14(0.23)	1.11(0.27)	<0.0001
Mould/dampness exposure (No. [%])	86(13.0%)	41(12.3%)	20(11.4%)	0.84	105(13.2%)	51(15.9%)	15(14.6%)	0.41
Current passive smoking exposure (No. [%])	353(53.3%)	172(51.7%)	112(63.6%)	0.02	446(55.9%)	191(59.5%)	67(65.0%)	0.16
Self-reported heavy traffic exposure (No. [%])	121(18.3%)	58(17.4%)	35(19.9%)	0.79	178(22.3%)	57(17.8%)	23(22.3%)	0.23

*Overweight (OW) and obese (O) children were defined following the gender- and age-specific cut-off points by Cole et Al[15]

Lung function data and personal exposures are presented as concerns non overweight-obese (non OW-O), overweight* (OW), and obese* (O) subjects. Differences in FVC, FEV_1_, FEF_25–75%_ (as percent of predicted), FEV_1_/FVC%, and FEF_25–75%_/FVC (as absolute percent) between males and females were evaluated by means of one-way ANOVA. Differences in frequency distribution of categorical variables were computed by χ^2^ test.

**Table 7:** Parameter estimated by multiple linear regression analysis models for FVC, FEV_1_, and FEV_25–75%_ as dependent variables and sex, age class, presence/ absence of personal history of respiratory symptoms, height, and weight as independent variables.

Dependent var.	FVC, R^2^ = .668			FEV_1_, R^2^ = .657			FEF_25–75%_, R^2^ = .333		
Parameter	β coefficient*	p value	95% confidence interval	β coefficient*	p value	95% confidence interval	β coefficient*	p value	95% confidence interval
Intercept	-4.152	.000	-4.455/-3.849	-3.788	.000	-4.055/-3.521	-4.069	.000	-4.656/-3.483
[Gender = F]	-.166	.000	-.191/-.141	-.059	.000	-.081/-.037	.182	.000	.134/.230
[Gender = M]	0^a^	.	.	0^a^	.	.	0^a^	.	.
[Age class ≤ 11]	-.148	.000	-.195/-.101	-.202	.000	-.243/-.160	-.413	.000	-.504/-.322
[Age class = 12]	-.124	.000	-.164/-.084	-.164	.000	-.199/-.129	-.324	.000	-.401/-.247
[Age class = 13]	-.055	.005	-.094/-.017	-.089	.000	-.123/-.055	-.191	.000	-.266/-.117
[Age class ≥ 14]	0^a^	.	.	0^a^	.	.	0^a^	.	.
[Respiratory symptoms = Yes]	.013	.368	-.016/.043	-.031	.019	-.057/-.005	-.149	.000	-.205/-.092
[Respiratory symptoms = No]	0^a^	.	.	0^a^	.	.	0^a^	.	.
Height (m)	4.452	.000	4.242/4.662	4.158	.000	3.973/4.343	4.971	.000	4.565/5.376
Weight (kg)	.009	.000	.007/.010	.004	.000	.003/.005	-.003	.035	-.005/.000

^a^This parameter is set to zero because it is redundant

*β coefficients indicate how much a dependent variable changes per each unit variation of the independent variable, taking into account the effect of the other independent variables in the model. For categorical variables, β coefficients represent the effect of moving from the “reference” category (^a^) to another

Data are shown for the overall sample of 3,200 children.

**Table 8:** Parameter estimated by multiple linear regression analysis models for FEV_1_/FVC and FEV_25–75%_/FVC as dependent variables and sex, age class, presence/ absence of personal history of respiratory symptoms, height, and weight as independent variables.

Dependent var.	FEV_1_/FVC, R^2^ = .135			FEF_25–75%_/FVC, R^2^ = .111		
Parameter	β coefficient*	p value	95% confidence interval	β coefficient*	p value	95% confidence interval
Intercept	87.856	.000	83.232/92.481	1.247	.000	1.038/1.456
[Gender = F]	2.998	.000	2.616/3.380	.125	.000	.107/.142
[Gender = M]	0^a^	.	.	0^a^	.	.
[Age class ≤ 11]	-2.339	.000	-3.058/-1.620	-.090	.000	-.123/-.058
[Age class = 12]	-1.686	.000	-2.291/-1.080	-.065	.000	-.093/-.038
[Age class = 13]	-1.244	.000	-1.832/-.656	-.047	.001	-.074/-.021
[Age class ≥ 14]	0^a^	.	.	0^a^	.	.
[Respiratory symptoms = Yes]	-1.360	.000	-1.808/-.913	-.053	.000	-.073/-.032
[Respiratory symptoms = No]	0^a^	.	.	0^a^	.	.
Height (m)	5.334	.001	2.135/8.534	.047	.527	-.098/.191
Weight (kg)	-.112	.000	-.131/-.094	-.004	.000	-.005/-.003

^a^This parameter is set to zero because it is redundant

*β coefficients indicate how much a dependent variable changes per each unit variation of the independent variable, taking into account the effect of the other independent variables in the model. For categorical variables, β coefficients represent the effect of moving from the “reference” category (^a^) to another

Data are shown for the overall sample of 3,200 children.

**Table 9:** Analysis performed on 807 children reporting wheeze, nocturnal cough, or exercise-induced cough.

	Non OW-O, (No. = 476)	OW, (No. = 226)	O, (No. = 105)	p value
FVC (% of predicted[SD])	96.0(11.3)	99.3(11.6)	99.9(11.2)	0.0003
FEV_1_ (% of predicted[SD])	99.1(11.3)	100.4(11.6)	99.8(10.8)	0.35
FEF_25–75%_ (% of predicted[SD])	100.4(21.7)	98.2(20.9)	95.1(18.9)	0.067
FEV_1_/FVC (%, mean[SD])	90.2(6.0)	88.0(6.1)	86.5(5.4)	<0.0001
FEF_25–75%_/FVC(L/s/L, mean[SD])	1.11(0.27)	1.03(0.25)	0.97(0.21)	<0.0001

*Overweight (OW) and obese (O) children were defined following the gender- and age-specific cut-off points by Cole et Al[15]

Mean lung function data are presented as concerns non overweight-obese (non OW-O), overweight* (OW), and obese* (O) subjects for FVC, FEV_1_, FEF_25–75%_ (as percent of predicted), FEV_1_/FVC%, and FEF_25–75%_/FVC (as absolute percent). P values of comparisons among non OW-O, OW, and O (one-way ANOVA) are shown.

## Discussion

In the present cross-sectional study, by evaluating 2,393 children without any personal history of wheeze, nocturnal cough or exercise-induced cough, we found that FVC and FEV_1_ appear to be positively correlated to weight in both males and females when corrected for sex, age, and height. Conversely, FEF_25–75%_ was not correlated to weight, while FEV_1_/FVC and FEF_25–75%_/FVC were negatively correlated to weight. These results produce further explanation for the identification of signs of respiratory impairment found in obese-overweight children without any history of obstructive respiratory disease [11,12].

### Relationship between lung function and weight

Checking the studies evaluating the effects of being overweight and obese on ventilatory function, it is notable that the relationship between lung function and BMI in children remains unclear [6,9], especially in healthy children. By a multiple regression model, we accounted for differences in age, gender, and height in the relationship between weight and respiratory variables. In this model, we found a non significant interaction between gender and weight: i.e., gender does not appear to influence the relationship between weight and respiratory variables, in agreement with a previous study by He et al, in which the positive correlation of FVC to BMI was significant in all children gender categories [18]. Conversely, Lang et al [19] found that obesity was associated with significantly reduced FEV_1_/FVC in males, while it was associated with improved lung function among females. In the latter study, performed in children with poorly controlled asthma, the significant decrease in FEV_1_/FVC ratio was obtained among obese males through a slight reduction in FEV_1_ and a slight increase in FVC. Among obese females, FEV_1_ and FVC increased and FEV_1_/FVC remained unchanged.

In each age class, the weight β coefficients for FVC were more elevated than the weight β coefficients for FEV_1_. This produced a disproportionate increase of FEV_1_ and FVC with increasing weight (Tables 3 and 7 and Fig 1): thus, the weight β coefficients for FEV_1_/FVC were negative (0.1 decrease per each kg of weight increase), and consequently FEV_1_/FVC resulted lower in OW and O subjects. Nevertheless, when small airways were evaluated by measuring FEF_25–75%_ [20], the weight β coefficient for FEF_25–75%_ was not significant. Similarly to the FEV_1_/FVC ratio, weight presented a negative β coefficient for FEF_25–75%_/FVC and this was significantly lower among both male and female OW-O subjects. The ratio between FEF_25–75%_ and FVC has been used as a surrogate measure of “dysanapsis”, reflecting the inequalities between the geometry of the tracheobronchial tree and the lung parenchyma [21]. Accordingly, a lower FEF_25–75_/FVC ratio was associated with higher airway sensitivity and reactivity to methacholine in susceptible subjects [22]. Our results support the hypothesis that, in children, the height corrected weight gain behaves as an index of body growth (that, in turn, produces an increase in lung volumes) [11]. Nevertheless, as weight increases, the disproportionate changes in FEV_1_ vs FVC and in FEF_25–75%_ vs FVC could cause a reduction of relative airway size (as expressed by using FEV_1_/FVC and FEF_25–75%_/FVC ratios as surrogate measures) at higher BMI values. Consequently, adolescents with a higher BMI may show proportionally narrower airways: this might, at least in part, contribute to the reported association between being overweight-obese and asthma [23].

### Effect of adiposity on airways and previous reports

Mechanical effects of adiposity are likely to occur in airways [24] and our data are in agreement with previous studies on children suggesting that higher BMI is associated with reduced lung function, as demonstrated by a lower FEV_1_/FVC ratio [25–27], and predicts higher asthma incidence in both sexes [28]. Consistently with our results, recently Han et al found that among children without asthma BMI was positively associated with FEV_1_ and FVC but inversely associated with FEV_1_/FVC [29]. Moreover, Davidson et al showed a positive correlation between FVC and BMI, while the relationship FEV_1_/BMI resulted not significant [30]. In the same paper, despite the fact that a negative correlation was found between expiratory reserve volume (ERV) and BMI, no significant change in total lung capacity was observed. Although no ERV measure was performed in the present work, in our opinion the quoted results further support the hypothesis of a mechanical effect of adiposity on airways with a redistribution of relevant lung volumes.

Interestingly, when we introduced the presence/absence of respiratory symptoms into the model, the β coefficients of all the variables remained unchanged. Moreover, when we repeated the analyses on symptomatic children, for each evaluated variable we found similar differences among non OW-O, overweight, and obese subjects. Thus, it seems that the effects of being overweight-obese on lung function may be independent from asthma-related airway obstruction. Because overweight and obese children showing reduced airflow may also have more asthma-like symptoms (primarily during physical exertion) [8], they may be affected by asthma overdiagnosis [23,31].

Our findings of significantly lower FEV_1_/FVC and FEF_25–75%_/FVC values in OW and O subjects are in line with the results of a longitudinal study performed on 654 young Australian adults (aged 27–36 years), first studied at the ages of 9, 12, or 15 years in which BMI and Lean Body Mass (LBM) were derived from anthropometric measures at baseline and at follow-up [32]. This study shows that the beneficial effect of increased BMI in childhood on adult FEV_1_ and FVC observed in previous longitudinal studies is likely to be attributable to larger childhood LBM and not to adiposity. Similarly, Tantisira et al showed that, along with an increase in spirometric indices, BMI increase was correlated to decrements in FEV_1_/FVC ratio, even in absence of asthma symptoms [33].

Adipose tissue is now recognized as a multifunctional organ with endocrine function through the production of different adipokines involved in inflammatory processes at different levels. In addition, obesity may cause chronic low-grade inflammation, contributing to systemic metabolic dysfunction associated with obesity-linked disorders [34]. Currently, this issue is still controversial. The mechanistic basis underlying the association between obesity and asthma has not been established, but many factors might contribute to it, such as mechanical factors, aspects of the systemic inflammation related to obesity including changes in energy-regulating hormones, comorbidities of obesity, or predisposition to common etiologies [35].

Strength of the present study is the evaluation of lung function in a large sample of children with a limited age-range. This allowed us to proceed in two steps. In the first step, we excluded subjects with a personal history of chronic respiratory symptoms: thus, we estimated the relationship between BMI and lung function without the interference of respiratory symptoms/diseases, also taking into account possible confounders such as domestic exposure to ETS, mould/dampness, and self-reported heavy traffic exposure. In the second step, as a sensitivity analysis, we repeated comparisons while including children with a personal history of allergic respiratory disease as well, ruling out a possible effect-modification on the physiological relationship between weight and lung function.

A possible limitation is the use of BMI as a proxy for adiposity. We are aware that BMI may not truly reflect adiposity, or give information on lean body mass. However, BMI is a simple epidemiological measure which is easily applied in large population studies, and it has recently demonstrated to correlate well to body fat mass in children and adolescents [36]. Moreover, despite none of the children enrolled for the study declared to be an active smoker, it is possible that some of them hid the truth for convenience: this, along with other potential confounding factors not included in the preliminary backward stepwise selection process, could have produced some interference in the analyses, even though all subjects presenting respiratory symptoms were excluded from the evaluation.

## Conclusions

Our study shows that both FVC and FEV_1_ are positively correlated to weight, when corrected for height. Nevertheless, due to a different magnitude in explanatory effect of weight on FVC and FEV_1_, the latter shows a disproportionate lower increase with weight gain with respect to FVC. Thus, at higher BMI, this phenomenon decreases both FEV_1_/FVC and FEF_25–75%_/FVC levels, independently from respiratory symptoms/diseases.
